# Transcriptome-Based Gene Modules and Soluble Sugar Content Analyses Reveal the Defense Response of Cotton Leaves to *Verticillium dahliae*

**DOI:** 10.3390/ijms252413326

**Published:** 2024-12-12

**Authors:** Shenglong Song, Yongtai Li, Yong Zhang, Feng Liu, Qian-Hao Zhu, Xinyu Zhang, Jie Sun, Yanjun Li

**Affiliations:** 1 The Key Laboratory of Oasis Eco-Agriculture, Agriculture College, Shihezi University, Shihezi 832000, China; songshenglong@stu.shzu.edu.cn (S.S.); liyongtai@stu.shzu.edu.cn (Y.L.); 20222012025@stu.shzu.edu.cn (Y.Z.); liufeng@shzu.edu.cn (F.L.); zhxy@shzu.edu.cn (X.Z.); 2 CSIRO Agriculture and Food, P.O. Box 1700, Canberra 2601, Australia; qianhao.zhu@csiro.au

**Keywords:** cotton, RNA-sequencing, *Verticillium dahlia*, soluble sugars

## Abstract

*Verticillium dahliae* is a soil-borne phytopathogenic fungus causing destructive Verticillium wilt disease that greatly threats cotton production worldwide. The mechanism of cotton resistance to Verticillium wilt is very complex and requires further research. In this study, RNA-sequencing was used to investigate the defense responses of cotton leaves using varieties resistant (Zhongzhimian 2, or Z2) or susceptible (Xinluzao 7, or X7) to *V. dahliae*. The leaf samples were collected at 48 and 72 hpi (hours post infection) from the two varieties infected by *V. dahliae* (strain Vd991) or treated by water. Compared to X7, Z2 had less genes responsive to *V. dahliae* infection at 72 hpi and had no DEGs (differentially expressed genes) at 48 hpi. WGCNA (Weighted Gene Co-Expression Network Analysis) revealed seven key gene modules which were responsible for the resistance of Z2 and susceptibility of X7. KEGG (Kyoto Encyclopedia of Genes and Genomes) analysis of these modules found that several reported disease resistance pathways were found to be up-regulated in Z2, with some of those pathways down-regulated in X7. Unexpectedly, several photosynthesis-related pathways were significantly up-regulated in the leaves of X7 infected by *V. dahliae*, leading to different profiles of glucose content, which was significantly decreased at 72 hpi and 48 hpi in X7 and Z2, respectively. These results suggest that the leaves of resistant varieties have a slower and different response to *V. dahliae* compared to those of the susceptible variety, as well as that the translocation of sugars produced by photosynthesis in cotton leaves might vary between the two varieties. Additionally, several HUB genes regulating disease response were identified, including NDR1/HIN1-like protein 12, DELLA protein, cytochrome P450 family protein and LRR receptor-like serine/threonine-protein kinase genes, which have been reported to be related to disease resistance in other plants, which might serve as potential candidates for breeding cotton disease resistance.

## 1. Introduction

Cotton is a vital economic crop worldwide, providing about 35% of the natural fibers used in the textile industry and serving as a significant source of edible oil and livestock feed [1]. Among the cotton species, *G. hirsutum* has the broadest distribution, accounting for 95% of global cotton production, and continues to be the primary focus of cotton breeding efforts [2]. Cotton plays a crucial role in China’s economy, especially in Xinjiang, the country’s largest cotton-producing region, where the cotton industry is a cornerstone of the local economy [3].

Cotton is often harmed by pathogens during its growth period. Verticillium wilt (VW), commonly known as the “cancer” of cotton, significantly affects both the yield and quality of cotton, resulting in substantial economic losses [4]. In China, losses attributed to Verticillium wilt in cotton account for 32.49% of the total losses caused by various diseases [5]. Therefore, overcoming Verticillium wilt in cotton production is of paramount significance. Currently, there are no effective biological or chemical control strategies for managing Verticillium wilt in cotton. Developing resistant cotton varieties through genetic engineering represents an effective, economical, and sustainable strategy for controlling Verticillium wilt, thereby playing a crucial role in sustaining global agricultural production. However, breeding disease resistant upland cotton varieties poses challenges due to limited genetic resources for Verticillium wilt resistance in cotton. Therefore, exploring new resistance mechanisms holds great significance for disease-resistant breeding.

Transcriptome analysis is an effective technique to study genome-wide gene expression changes in plants and has been used extensively to identify potential genes and molecular mechanism underling various stresses [6,7,8]. Verticillium wilt is caused by *Verticillium dahliae*, which enters into cotton through roots. Therefore, the infected cotton roots have been widely used for transcriptomic analysis. The phenylpropanoid biosynthesis pathway was activated in cotton roots infected with *V. dahliae* [9]. Root transcriptome analysis revealed that genes associated with protein serine/threonine kinase, peroxidase enzyme activity, plant hormone signal transduction, responses to biotic and abiotic stress, and lignin metabolism are crucial for cotton disease resistance [10]. Genes related to the plant signal transduction pathway were found to be enriched in disease-resistant varieties through root transcriptome analysis [11]. After several days of infection with *V. dahliae*, the cotton leaves turn yellow, wilt or even fall off [12]. Although the cotton leaves do not show any disease symptoms in the few days after infection, gene expression may have changed. However, the changes in gene expression in cotton leaves after infection by *V. dahliae* are still unclear.

Leaves are organs that produce sugars through photosynthesis. The sugars produced by photosynthesis in the mesophyll cells of mature leaves are not only used for plant metabolism but also transported to various sink tissues for storage and utilization. In the interaction between plant and pathogens, pathogens obtain energy (sugars) from plants, accompanied by a redistribution of sugar content and types within the plant body. For example, in wheat and rice, plant pathogens have the ability to alter the transport and distribution of different sugars in host tissues [13,14]. Increased glucose transport is observed in wheat infected with powdery mildew [15]. Infection by pathogens can enhance the efflux of sugars and also lead to the accumulation of sucrose and hexose in the growth sites of pathogens, which can be used for their growth [16]. After 48 h of infection by *V. dahliae*, cotton plants show a significant increase in glucose content in their roots [17]. However, there is still limited research on the redistribution of sugar content in cotton during pathogen infection.

Based on the studies on wheat and rice [13,14], we hypothesize that infection by *V. dahliae* leads to differential sugar redistribution in cotton leaves, which may contribute to the plant’s disease resistance or susceptibility. To verify this hypothesis, we analyze the changes in sugar content in leaves after 48 h and 72 h of infection by GC-MS (Gas Chromatography–Mass Spectrometry). The changes in sugar content are inevitably linked to the changes in gene expression. Therefore, transcriptome sequencing is also conducted to compare the difference in gene expression in leaves between resistant and susceptible cotton varieties after 48 h and 72 h of infection and to provide molecular evidence for the changes in sugar content. Unlike most studies that focused on infected cotton roots, this study redirects its focus to the alterations within the leaves, potentially offering novel perspectives. This study broadens our understanding of the molecular mechanisms of cotton disease resistance.

## 2. Results

### 2.1. Characterization of Each Cotton Variety’s Response to V. dahliae Infection

**Figure 1:**
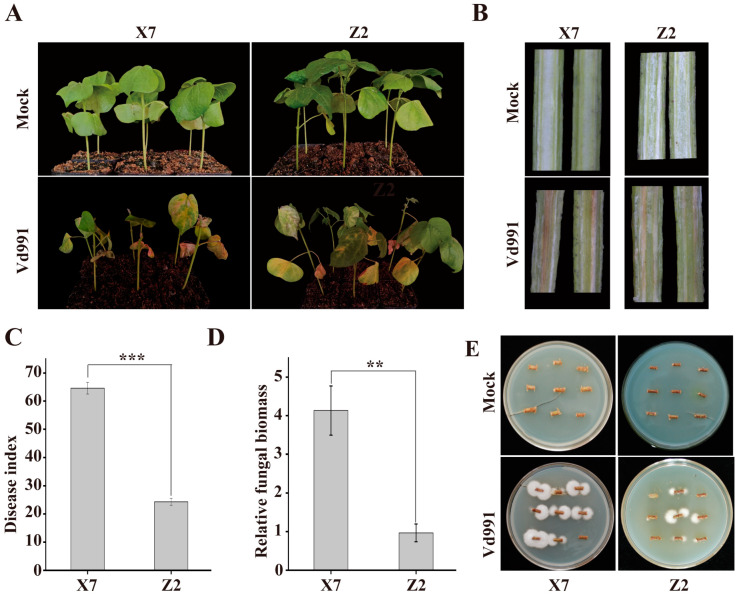
Investigation of disease development in X7 and Z2 after infection by Vd991. Mock means cotton treated with water: (**A**) disease symptoms of X7 and Z2 at 14 dpi; (**B**) symptoms of vascular bundle in X7 and Z2 at 14 dpi; (**C**) Disease Index of X7 and Z2 at 14 dpi; (**D**) accumulation of fungal biomass in X7 and Z2 at 14 dpi measured by qRT-PCR; (**E**) fungal recovery from stems of X7 and Z2 at 14 dpi. The data represent the mean values ± standard deviations of three independent biological replicates, and the statistical method used was the T-test (** *p* < 0.01; *** *p* < 0.001).

X7 and Z2 have been reported as susceptible and resistant varieties [18,19,20], respectively. In this study, we planted the two varieties under the same conditions and compared their resistance to *V. dahliae* (strain Vd991). It was found that X7 showed more severe yellowing, wilting and shedding of leaves, as well as more obvious browning of vascular bundles, compared to Z2 (Figure 1A,B). The Mock (water treatment) plants of two varieties showed no disease symptoms (Figure 1A,B). The Disease Index of X7 was significantly higher than that of Z2 at 14 dpi (Figure 1C). Fungal biomass detection and recovery assays revealed significantly higher fungal biomass in infected X7 compared to Z2 (Figure 1D,E). These results confirmed that X7 is susceptible to *V. dahliae* and Z2 is resistant to this pathogen.

### 2.2. Overview of the Transcriptome Sequencing Results

For transcriptome profiling, leaf samples were collected at 48 and 72 h post inoculation (hpi) from three replicates of both X7 and Z2, with water treatment (Mock) as the control, to construct 24 cDNA libraries. The infected X7 samples at 48 and 72 hpi were designated as X7-48h-T and X7-72h-T, and the infected Z2 samples were designated as Z2-48h-T and Z2-72h-T, respectively. The water-treated X7 samples at 48 and 72 hpi were designated as X7-48h-M and X7-72h-M, and the water-treated Z2 samples were designated as Z2-48h-M and Z2-72h-M, respectively (Table 1). In total, 164.25 Gb of raw data were acquired, from which 158.21 Gb of high-quality clean data were obtained after the removal of low-quality reads. The Q20 proportion ranged from 98.30% to 98.71%, while the Q30 proportion ranged from 94.56% to 95.66%. Additionally, the GC content ranged from 43.75% to 44.73%. The high-quality clean reads were aligned to the upland cotton genome, yielding a mapping proportion (proper map) of more than 98.15% and a proportion of uniquely mapped reads (unique map) of more than 92.61% (Table 1). The reliability of the RNA-seq results was further validated by the qRT-PCR analysis of randomly selected genes, demonstrating strong concordance between RNA-seq and qRT-PCR (Figure S1).

### 2.3. Identification of DEGs (Differentially Expressed Genes) in X7 and Z2 Following V. dahliae Infection

**Figure 2:**
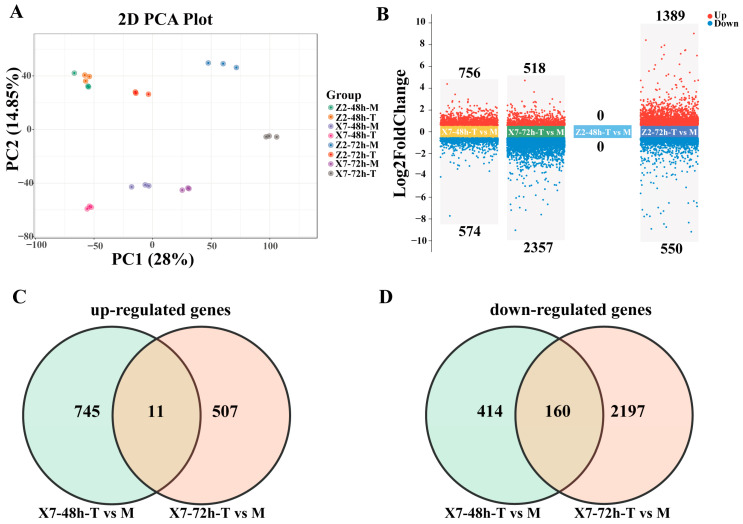
The identification of differentially expressed genes (DEGs) in X7-T vs. Mock and Z2-T vs. Mock comparisons: (**A**) PCA score plots with each point representing an independent biological replicate. (**B**) Volcanic plots of DEGs in X7-T vs. Mock and Z2-T vs. Mock comparisons. Red and blue points represent the up-regulated and down-regulated DEGs, respectively. (**C**) Venn diagram of up-regulated DEGs in X7-T vs. Mock comparisons. (**D**) Venn diagram of down-regulated DEGs in X7-T vs. Mock comparisons. Capital ‘T’ represents Treatment, and ‘M’ represents Mock.

To explore the changes in gene expression in the leaves of X7 and X2 during fungal infective process, we compared the dynamic changes in the transcriptomes of infected X7 and X2 at 48 and 72 hpi to those of the water treatments (Mock). As shown in Figure 2A, the samples of the two varieties under the two treatments (water and infection treatments) were scattered, while the samples in each treatment were clustered, indicating that the samples had good repeatability and that the responses of the two varieties to *V. dahliae* were significantly different. Comparing X7-48h-T to X7-48h-M and X7-72h-T to X7-72h-M, 1130 DEGs (including 756 up-regulated genes and 574 down-regulated genes) and 2875 DEGs (consisting of 518 up-regulated genes and 2357 down-regulated genes) were identified, respectively (Figure 2B). In the comparisons of Z2-48h-T vs. Z2-48h-M and Z2-72h-T vs. Z2-72h-M, 0 DEGs and 1939 DEGs (comprising 1389 up-regulated genes and 550 down-regulated genes) were identified, respectively (Figure 2B). Although the disease symptoms appeared on the leaves after about 10 days of infection, there was a significant change in gene expression in the leaves of the susceptible variety (X7) at 48 hpi. The resistant variety (Z2) exhibited fewer differentially expressed genes (DEGs) compared to the susceptible variety (X7) at both 48 and 72 hpi, indicating a slower response to *V. dahliae* infection in Z2.

### 2.4. KEGG Pathway Enrichment Analyses of DEGs in Leaves of X7 and Z2 After Infection by V. dahliae

**Figure 3:**
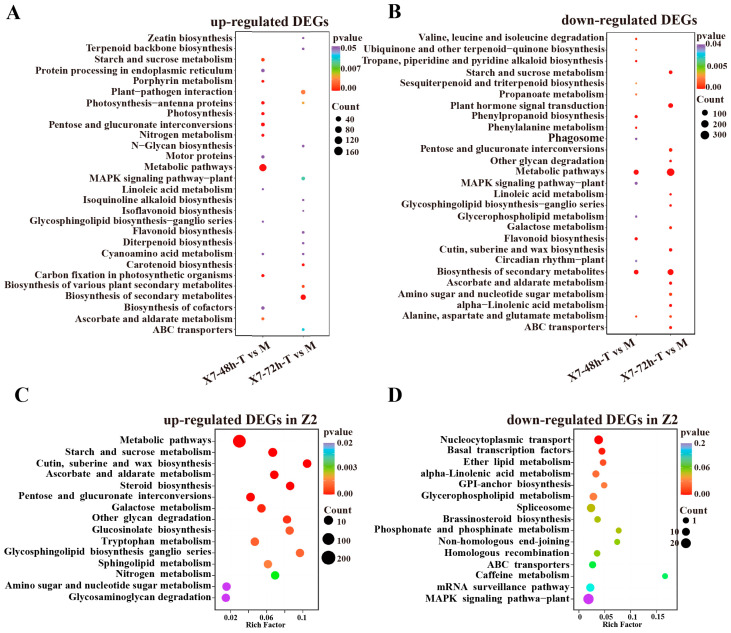
KEGG pathway enrichment analyses of the DEGs in X7 and Z2 after infection by *V. dahliae*. (**A**) KEGG pathway enrichment analysis of the up-regulated DEGs in X7; (**B**) KEGG pathway enrichment analysis of the down-regulated DEGs in X7; (**C**) KEGG pathway enrichment analysis of the up-regulated DEGs in Z2; (**D**) KEGG pathway enrichment analysis of the down-regulated DEGs in Z2.

A total of 1263 up-regulated DEGs were identified in X7, with 745 differentially expressed at 48 hpi, 507 at 72 hpi, and 11 at both time points (Figure 2C). A total of 2771 down-regulated DEGs were identified in X7, with 414 differentially expressed at 48 hpi, 2197 at 72 hpi, and 160 at both time points (Figure 2D). KEGG enrichment analysis showed that the up-regulated DEGs were highly enriched in photosynthesis and carbon metabolic pathways, such as photosynthesis-antenna proteins, nitrogen metabolism, pentose and glucuronate interconvers, photosynthesis, carbon fixation in photosynthetic organisms, porphyrin metabolism, starch and sucrose metabolism, and carotenoid biosynthesis (Figure 3A). The down-regulated DEGs in X7 were highly enriched in the well-known disease resistance pathways, such as flavonoid biosynthesis, the biosynthesis of secondary metabolites, phenylpropanoid biosynthesis, phenylalanine metabolism, and plant hormone signal transduction (Figure 3B).

For Z2, KEGG enrichment analysis showed that the 1389 up-regulated DEGs at 72 hpi were highly enriched in metabolic pathways, starch and sucrose metabolism, cutin, suberine and wax biosynthesis, ascorbate and aldarate metabolism, steroid biosynthesis, pentose and glucuronate interconversions, galactose metabolism and other glycan degradation pathways (Figure 3C). The 550 DEGs down-regulated at 72 hpi were highly enriched in nucleocytoplasmic transport and basal transcription factors (Figure 3D). Compared with X7, no photosynthesis-related pathway was found to be up-regulated in Z2. Disease resistance-related pathways were down-regulated in X7, again suggesting that the responses of the two varieties to *V. dahliae* in leaves were significantly different.

### 2.5. WGCNA Analysis of the Transcriptomes Responding to V. dahliae Infection

**Figure 4:**
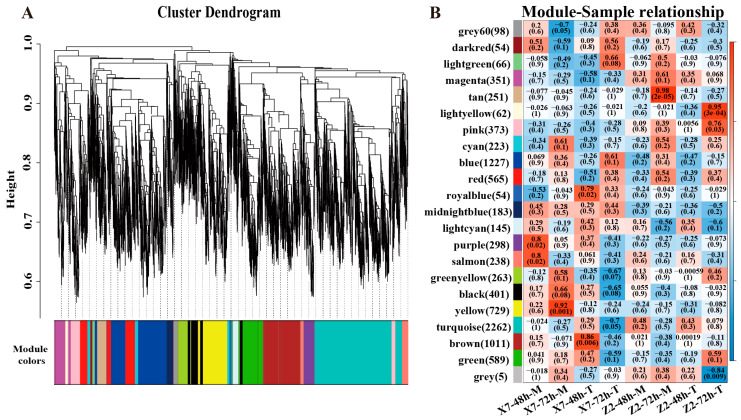
WGCNA analysis of key modules related to the *V. dahliae* responses in the two varieties. (**A**) Clustering dendrogram of co-expression modules. Each leaf in the tree represents one gene. The major tree branches constitute 20 modules labeled by different colors. The *x*-axis represents genes, and the *y*-axis represents the co-expression distance. Modules are identified using dynamic tree cutting by dividing the dendrogram at significant branch points. The horizontal bar immediately below the dendrogram represents modules indicated by different colors. (**B**) Module–sample point correlation. Each row represents a module, and each column corresponds to a specific sample. The color of each cell at the row–column intersection indicates the correlation coefficient between the module and the sample. Red indicates a high degree of correlation between a specific module and the sample.

WGCNA analysis was subsequently undertaken to explore the co-expression networks of the DEGs using 9948 DEGs, including all the DEGs in X7-48h-T vs. X7-48h-M, Z2-48-T vs. Z2-48h-M, X7-72h-T vs. X7-72h-M, Z2-72-T vs. Z2-72h-M, X7-48h-T vs. X7-72h-T, X7-48h-M vs. X7-72h-M, Z2-48h-T vs. Z2-72h-T, and Z2-48h-M vs. Z2-72h-M comparisons (Figure S2). A total of 20 co-expression modules were identified based on gene expression similarities (Figure 4A). A module–sample point correlation heatmap clearly exhibited the correlation between each module and each sample (Figure 4B). Eight modules (tan, light yellow, pink, royal blue, purple, salmon, yellow and brown) showed a correlation coefficient > 0.7 in at least one sample. DEGs up-regulated in infected X7 (susceptible variety) were mainly included in the royal blue (54) and brown (1011) modules, while DEGs up-regulated in Z2 (resistant variety) were mainly included in the light yellow (62) and pink (373) modules. DEGs down-regulated in X7 infected with *V. dahliae* were mainly included in the purple (298), salmon (238) and yellow (729) modules, while those in Z2 were mainly included in the tan (251) modules. The up-regulated DEGs included in the light yellow and pink modules of Z2 were considered to be responsible for the resistance of Z2 to *V. dahliae*, while the up-regulated DEGs included in the royal blue and brown modules and the down-regulated DEGs in the purple, salmon and yellow modules of X7 were considered to be responsible for the susceptibility of X7 to *V. dahliae*.

### 2.6. Gene Modules Related to the Resistance of Z2 and Susceptibility of X7 to V. dahliae

**Figure 5:**
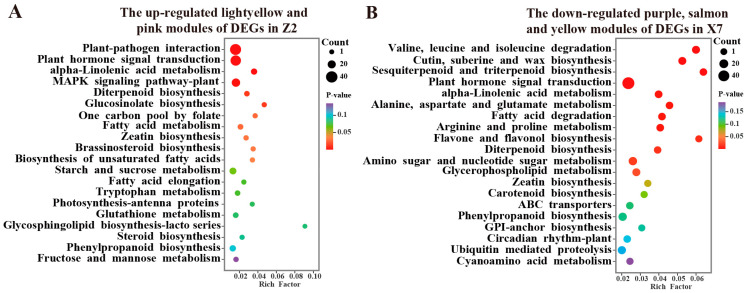
Analyses of the gene modules up-regulated in Z2 (light yellow and pink) and down-regulated in X7 (purple, salmon and yellow): (**A**) KEGG enrichment analysis of DEGs in the modules up-regulated in Z2; (**B**) KEGG enrichment analysis of DEGs in the modules down-regulated in X7.

To explore the molecular mechanisms underlying the resistance of Z2 to Verticillium wilt, we conducted KEGG analyses using all the genes of the light yellow and pink modules, and we found that genes in the two modules were highly enriched in the plant–pathogen interaction, plant hormone signal transduction, alpha-Linolenic acid metabolism, and MAPK signaling pathway–plant pathways (Figure 5A). The DEGs in these pathways were all up-regulated after induction by *V. dahliae* (Figure S3). Additionally, a total of 38 genes were found to be involved in the plant–pathogen interaction pathway, including several WRKY transcription factor and protein kinase genes (Table S1).

To explore the molecular mechanisms underlying the differences between Z2 and X7 in resistance to Verticillium wilt, we also conducted KEGG analyses on the modules up- and down-regulated in X7 and found that the down-regulated genes in the purple, salmon and yellow modules of X7 were highly enriched in some pathways that have been found to be up-regulated in Z2, such as plant hormone signal transduction and alpha-Linolenic acid metabolism, and those genes were also enriched in several well-known disease resistance pathways, such as flavone and flavonol biosynthesis and cutin, suberine and wax biosynthesis (Figure 5B). The DEGs in these pathways were all down-regulated after induction with *V. dahliae* (Figure S3). These pathways being down-regulated in X7 might be responsible for the susceptibility of X7 to *V. dahliae*

### 2.7. Energy- and Substance Metabolism-Related Pathways

**Figure 6:**
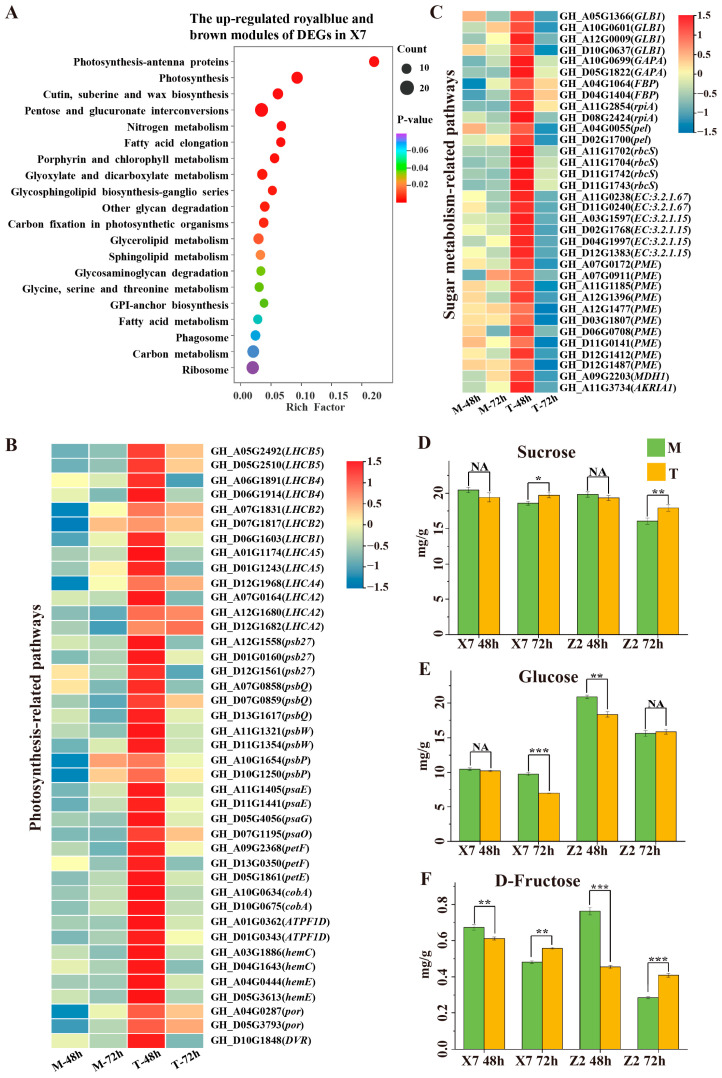
Analyses of the gene modules up-regulated in X7 (royal blue and brown) and the sugar content in X7 and Z2 leaves after infection by *V. dahliae*: (**A**) KEGG enrichment analysis of the DEGs in modules up-regulated in X7 (royal blue and brown); (**B**,**C**) a heatmap showing the expression of the DEGs in modules up-regulated in X7 (royal blue and brown); (**D**–**F**) the contents of sucrose, glucose, and fructose in X7 and Z2 leaves from *V. dahliae*-infected and water-treated plants. The data represent the mean values ± standard deviations of three independent biological replicates, and the statistical method used was the T-test (* *p* < 0.5; ** *p* < 0.01; *** *p* < 0.001; NA means *p* > 0.5).

It was notable that up-regulated genes in the royal blue (54) and brown (1011) modules of X7 were highly enriched in the photosynthesis pathway, including photosynthesis-antenna proteins, photosynthesis, and porphyrin and chlorophyll metabolism, and those genes were also enriched in some sugar metabolism-related pathways, including pentose and glucuronate interconversions, other glycan degradation, and carbon fixation in photosynthetic organisms (Figure 6A). A total of 41 up-regulated DEGs were involved in the photosynthetic-related pathway, including 17 photosystem II genes (such as *psbP*, *psbQ*, *psbW*, *psb27*, *LHCB1*, *LHCB2*, *LHCB4* and *LHCB5*), 10 photosystem I genes (such as *psaE*, *psaG*, *psaO*, *LHCA2*, *LHCA4* and *LHCA5*), 3 photosynthethic electron transport genes (such as *petE* and *petF*), 2 F-type ATPase genes (such as *ATPF1D*), and 9 genes related to porphyrin and chlorophyll metabolism (*cobA*, *hemC*, *hemE*, *por* and *DVR*). All of the photosynthesis-related genes were up-regulated in X7 at 48 hpi (Figure 6B).

The sugar metabolism-related genes in the two up-regulated modules (royal blue and brown) included 4 beta-galactosidase (*GLB1*), 2 glyceraldehyde-3-phosphate dehydrogenase (*GAPA*), 2 fructose-1,6-bisphosphatase I (*FBP*), 2 ribose 5-phosphate isomerase A (*rpiA*), 2 pectate lyase (*pel*), 4 ribulose-bisphosphate carboxylase small chain (*rbcS*), 2 galacturan 1,4-alpha-galacturonidase (*EC:3.2.1.67*), 4 polygalacturonase (*E3.2.1.15*), 10 pectinesterase (*PME*), 1 malate dehydrogenase (*MDH1*), and 1 alcohol dehydrogenase genes (*AKR1A1*). Some genes of these two modules were related to the pectin degradation and galactose metabolisms, such as *PME*, *pel*, *EC:3.2.1.67*, *E3.2.1.15* and *GLB1*. The expression of these carbon metabolism-related genes was all up-regulated in X7 at 48 hpi (Figure 6C).

Plants make sugars through photosynthesis. The significant up-regulation of photosynthesis-related genes let us speculate that the sugar content in leaves might have been changed. We therefore measured the contents of the three main soluble sugars (sucrose, glucose, and fructose) in X7 leaves from both treatments (*V. dahliae* infection and water treatment). It was found that the content of sucrose at 72 hpi was significantly higher in the leaves from plants infected by *V. dahliae* than in the leaves of the Mock treatment, while no difference was found between the two treatments at 48 hpi (Figure 6D). Upon *V. dahliae* infection, the content of glucose did not change at 48 hpi but was significantly reduced at 72 hpi (Figure 6E). *V. dahliae* infection significantly reduced the fructose content at 48 hpi but significantly increased the fructose content at 72 hpi (Figure 6F). The increased sucrose and fructose contents at 72 hpi might have been due to the increase in the expression level of photosynthesis-related genes at 48 hpi. The reduction in glucose at 72 hpi was possibly due to the massive translocation of photosynthetic products from the leaves to the site of infection and sinks. The high expression of *FBP* genes at 48 hpi may have increased the utilization of the glycolytic pathway for fructose, leading to a decrease in fructose content. To compare the differences in sugar content changes between Z2 and X7 upon *V. dahliae* infection, we also measured the contents of the three sugars (sucrose, glucose, and fructose) in the Z2 leaves collected from *V. dahliae*-infected and uninfected plants. For sucrose and fructose, a pattern similar to that observed in X7 was also evident in Z2. But, for glucose, the pattern was different between Z2 and X7, with a significant reduction being observed at 48 hpi but not at 72 hpi (Figure 6E).

### 2.8. HUB Genes Contribute to Cotton’s Resistance to V. dahliae

**Figure 7:**
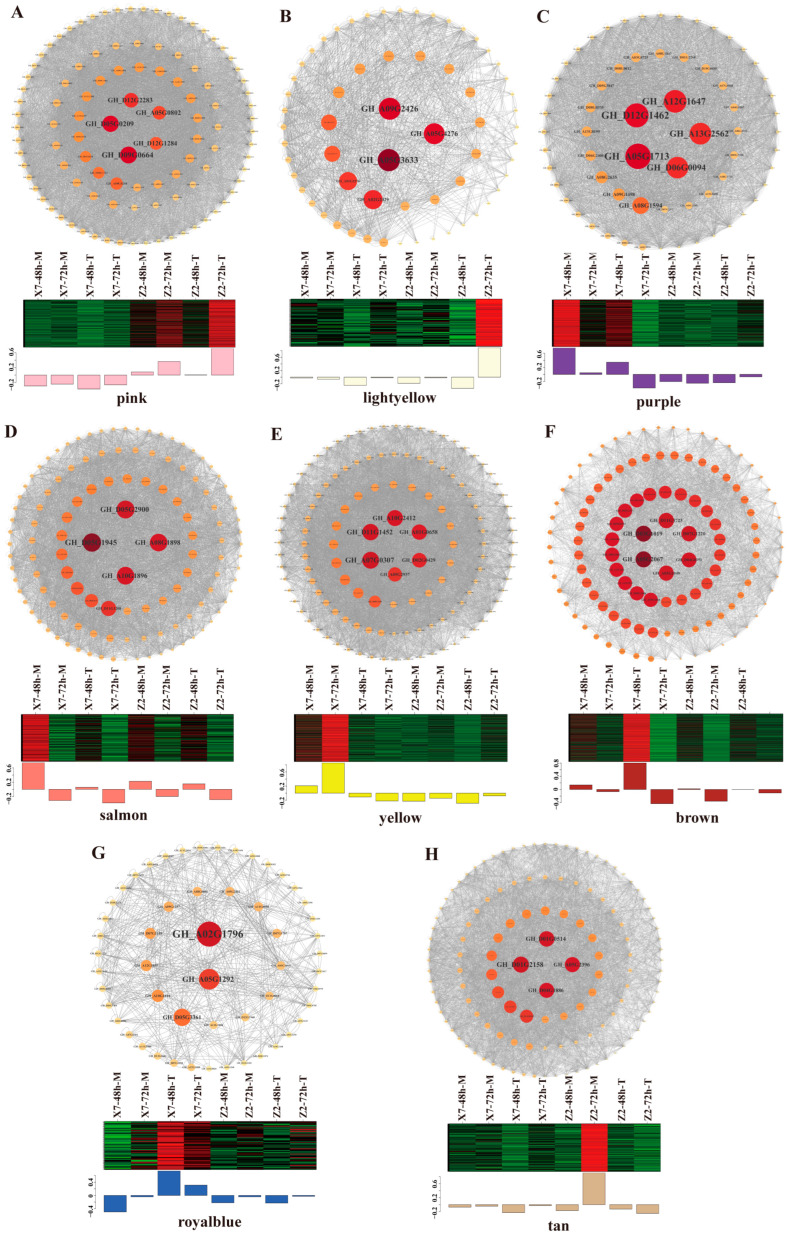
The co-expression network and expression profile of the genes within the pink, light yellow, purple, salmon, yellow, brown, royal blue, and tan modules: (**A**–**H**) Upper: co-expression network of DEGs in each module. Lower: heatmap showing the expression profiles of the genes in each module.

The genes from the pink, light yellow, purple, salmon, yellow, brown, royal blue, and tan modules were selected, and we analyzed their edges and nodes using the Metware Cloud (https://cloud.metware.cn). Genes with a correlation coefficient greater than 0.8 were selected for plotting, and the gene-network visualization was created using Cytoscape (Figure 7A–H). As a result, a total of 35 HUB genes were obtained (Table 2), including several reported disease resistance genes, such as NDR1/HIN1-like protein 12 (GH_A05G0802), DELLA protein (GH_D05G0209), cytochrome P450 family protein (GH_D09G0664), LRR receptor-like serine/threonine-protein kinase FLS2 (GH_A10G2412), natural resistance-associated macrophage protein 2 (GH_A02G1796), LRR receptor-like serine/threonine-protein kinase ERECTA (GH_A05G2396), and calcium-binding protein CML genes (GH_D01G2158). Additionally, we found several sugar metabolism-related genes to be hub genes, such as carbon catabolite-derepressing protein kinase (GH_A09G2426), beta-1,3-galactosyltransferase (GH_A05G1713), beta-1,4-mannosyl-glycoprotein beta-1,4-N-acetylglucosaminyltransferase (GH_A12G1647), and 1,4-beta-D-xylan synthase (GH_D12G1462) genes. Among these HUB genes, only a few were connected to the KEGG pathways, as previously described. For example, GH_A10G2412 and GH_A05G2396 were in the MAPK signaling pathway–plant pathway, GH_D05G0209 and GH_A05G2067 were in plant hormone signal transduction pathway, and GH_A10G2412 and GH_D01G2158 were in plant–pathogen interaction pathway.

## 3. Discussion

### 3.1. The Differential Regulation of Disease Resistance Pathways in Z2 and X7 Explains Their Contrasting Resistance to V. dahliae

Previous transcriptomic analyses found that starch and sucrose metabolism, pentose and glucuronate in terconversions, the MAPK signaling pathway, flavonoid biosynthesis, phenylpropanoid biosynthesis, and plant hormone signal transduction pathways were significantly enriched in cotton roots in response to *V. dahliae* infection [9,21]. The flavonoid biosynthesis pathway contributed positively to cotton resistance against *V. dahliae* [22,23]. Genes in phenylpropanoid biosynthesis pathway have been proved to be involved in cotton resistance to *V. dahliae* [9,24,25]. In this study, WGCNA combined with KEGG analysis found that plant hormone signal transduction and the MAPK signaling pathway were significantly up-regulated in leaves of Z2 (resistant variety), consistent with the transcriptomic findings in cotton roots.

Compared with Z2, X7 (susceptible variety) had no disease resistance pathways up-regulated in leaves, while the plant hormone signal transduction and flavonoid biosynthesis pathways were down-regulated in leaves of X7. Additionally, we found that the plant–pathogen interaction and alpha-Linolenic acid metabolism pathways were up-regulated in leaves of Z2 but down-regulated in leaves of X7. The plant–pathogen interaction pathway plays an important role in the plant disease resistance process [26,27]. The alpha-Linolenic acid metabolism produces signaling molecules such as jasmonic acid, which was involved in the defense responses of plants and regulates their resistance to diseases [28,29]. The disease resistance pathways up-regulated in Z2 and down-regulated in X7 lead to the differences in disease resistance between the two varieties. While disease resistance pathways were up-regulated in Z2, we also observed unexpected changes in photosynthesis-related pathways in X7, which may contribute to its susceptibility.

### 3.2. Photosynthesis-Related Pathways Up-Regulated in X7 Were Also Responsible for the Susceptibility of X7 to V. dahliae

After infection by *V. dahliae*, the cotton leaves exhibit yellowing and wilting, and the photosynthesis decreases, leading to reduced yield [30]. Unexpectedly, we observe a significant up-regulation of photosynthesis-related pathways, including photosynthesis-antenna proteins, photosynthesis and porphyrin and chlorophyll metabolism, in X7 (susceptible variety) after infection by *V. dahliae*. In plants, the source tissue produces photosynthetic products, while the sink tissue imports and uses them [31,32]. The coordination of source–sink relationship is an indispensable basis for plant growth. Pathogens can disrupt the mutual relationship between source and sink in plants, destroy the plant’s own defense system through various mechanisms, and obtain carbon nutrition from the sink cells for their own growth to complete their life cycle [33,34]. Therefore, the up-regulation of photosynthesis-related genes might be driven by the utilization of carbon sources by pathogens to enhance their growth and pathogenesis, which might contribute to the susceptibility of X7 to *V. dahliae.*

The up-regulation of photosynthesis-related pathways might lead to changes in the soluble sugar content in leaves. GC-MS analysis found that *V. dahliae* infection increased the sucrose content at 72 hpi in both varieties and decreased the glucose content at 72 hpi and 48 hpi in X7 and Z2, respectively. And the D-fructose content showed a trend of first decreasing and then increasing in both varieties upon *V. dahliae* infection. These results suggest that *V. dahliae* infection might cause the translocation of sugars produced by photosynthesis in cotton leaves. In the disease-resistant variety Z2, the up-regulated DEGs were not obviously enriched in photosynthesis-related pathway. Due to the similar changes in sugar content between Z2 and X7, we speculated that the resistant variety might also present sugar translocation after infection by *V. dahliae*. It was notable that fewer DEGs were observed in leaves of the resistant variety (Z2) at both 48 and 72 hpi compared to X7, and no DEGs were observed in the leaves of the disease resistant variety (Z2) at 48 hpi, indicating that the leaves of the resistant variety (Z2) have a slower response to *V. dahliae* compared to the susceptible variety (X7), which might be the reason why we did not observe photosynthesis-related pathways in Z2 at 48 or 72 hpi.

### 3.3. HUB Genes Can Serve as Candidates for Cotton Disease Resistance Breeding

Our co-expression network analysis on different modules identified several HUB genes. Among the HUB genes identified in the up-regulated modules in Z2, several have been reported to be involved in plant disease resistance, such as the NDR1/HIN1-like (NHL), DELLA protein and cytochrome P450 family protein genes. The roles of these genes in plant disease resistance have been confirmed in other crops. The overexpression of *GmNHL1* and *GmNHL8* of *Glycine max* in *A. thaliana* plants was found to enhance resistance to *Heterodera glycines* by altering the signal in the jasmonic acid (JA) and ethylene (ET) pathways [35]. In *Solanum tuberosum*, the overexpression of *StPOTHR1* was reported to enhance resistance against *Phytophthora infestans* [36]. In *Capsicum annuum*, a genome-wide analysis of the NHL gene family revealed that *CaNHL4* could play a role in resistance to *P. capsici*, *P. syringae* and *TMV* infection [37]. DELLA proteins were found to influence plant disease resistance through gibberellin acid (GA). The disease resistance of cassava was reduced after the silencing of *MeDELLA* [38]. Cytochrome P450 family proteins (CYPs) were involved in the signaling pathways that were triggered by jasmonic acid and methyl jasmonate. Methyl jasmonate was known to activate the *CYP82A3* gene in soybeans upon fungal infections. A *N. benthamiana* transgenic line overexpressing *CYP82A3* showed highly resistance to gray mold and black shank diseases [39]. IQ domain-containing protein played a critical role in basic host defenses [40,41]. Among the HUB genes from the down-regulated module in Z2, the calcium-binding protein CML was related to the peanut’s response to the stimulation of *Ralstonia solanacearum* [42].

Several HUB genes from the down-regulated module in X7 were related to disease resistance, e.g., deubiquitinase DeSI2, LRR receptor-like serine/threonine-protein kinase FLS2 and transcription factor genes. The roles of these genes in plant disease resistance have been confirmed in other crops. The overexpression of *DeSI2* in potato led to increased resistance to *P. infestans*, while silencing *StDeSI2* resulted in the down-regulation of a set of defense-related genes [43]. LRR receptor-like serine/threonine-protein kinase FLS2 was a pattern recognition receptor in plants, In soybeans, the knockout of *GmFLS2* enhanced disease symptoms [44]. Transcription factor bHLH147-bHLH was reported to positively regulate resistance to *V. dahliae* in upland cotton. The overexpression of the bHLH transcription factor *GhPAS1* in ZM24 significantly ameliorated the infection symptoms [45]. In view of these reported results, some of the HUB genes identified in this study might serve as potential candidates for cotton disease resistance breeding.

## 4. Materials and Methods

### 4.1. Plant and Fungus Material and Culture Conditions

Two upland cotton (*G. hirsutum*) varieties, Xinluzao 7 (X7), which is susceptible to *V. dahliae*, and Zhongzhimian 2 (Z2), which is resistant to *V. dahliae*, were used in this study. The cotton seeds were sterilized with alcohol, evenly placed in germination boxes covered with moist gauze, and subsequently incubated in a 28 °C incubator. Upon germination, the seeds were planted in a soil mixture with a 3:1 ratio of nutrient soil and vermiculite and placed in a growth chamber at 28 °C with a relative humidity of 60% and a photoperiod of 16 h light/8 h dark.

The highly virulent V991 strain of *V. dahliae* used in this study was confirmed and provided by the Institute of Cotton Research of Chinese Academy of Agricultural Sciences (Anyang, China). The strain was cultured in liquid Czapek’s medium and incubated at 25 °C for 5 days. The conidia were collected by filtering liquid culture through 8 layers of sterile gauze and were then diluted to 1 × 10^7^ CFU/mL with sterile water. Cotton seedlings with two true leaves were inoculated with a conidial suspension (1 × 10^7^ CFU/mL) via root irrigation [46].

### 4.2. Characterization of Disease Symptoms of Cotton Varieties

At 14 dpi (days post infection), the disease symptoms, including the yellowing and wilting of leaves and browning of xylem, were recorded. The Disease Index (DI) was calculated using a five-grade (0, 1, 2, 3, and 4) classification of disease symptoms. The formula for calculating DI was Disease Index = Σ (Diseased plant count × Grade number)/(Total number of inspected cotton plants × 4) × 100. For fungal recovery assay, 10 infected seedlings were randomly selected, and their stems were collected and cut into 2 cm long segments, which were sterilized with 84 disinfectant liquid for 10 min and then rinsed 3–5 times with sterilized ddH_2_O. The sterilized stem segments were evenly placed on PDA medium containing cefotaxime (0.2 mg/mL) and incubated at 25 °C in the dark for fungal growth observation after 7 days of culture. For fungal biomass detection, 2 cm stem segment above cotyledon node were taken from infected seedlings and used for genomic DNA extraction with the CTAB method. The Verticillium wilt pathogen-specific primers ITS1-F and ST-Vel-R [47] were used for qRT-PCR (real-time quantitative PCR). The cotton *GhUBQ7* gene (DQ116441.1) was used as an internal reference. The qRT-PCR assays were performed with PerfectStart Green qPCR SuperMix (TransGen Biotech, Beijing, China) on a LightCycler 480II instrument (Roche, Indianapolis, IN, USA), and the results were analyzed using the 2^−ΔΔCT^ method.

### 4.3. RNA Sequencing (RNA-Seq) Analysis

Cotton leaves were collected from two cotton varieties infected with Vd991 and uninfected controls (treated with water) at 48 h and 72 hpi (hours post infection). Total RNA was extracted using the ethanol precipitation and CTAB-PBIOZOL methods [48]. The Hieff NGS^®^ Ultima Dual-mode mRNA Library Prep Kit (Yeasen, Shanghai, China) was used to construct the transcriptome library. RNA sequencing was conducted, utilizing the Illumina Novaseq 6000 platform, by Wuhan Meiteweier Biotechnology Co., Ltd. (www.metware.cn, Wuhan, China). Clean reads obtained were aligned to the cotton reference genome TM-1_V2.1 (http://cotton.zju.edu.cn/download.html. accessed on 3 January 2024) using HISAT2_V2.2.1 [49] software. Subsequently, the matched clean reads were assembled into transcripts using StringTie_V2.1.6 software based on their positions on the reference genome [50].

### 4.4. Identifying Differentially Expressed Genes (DEGs) and GO and KEGG Enrichment Analyses

Fragments Per Kilobase of transcript per Million mapped fragments (FPKM) was utilized as a metric for quantifying gene expression levels. The DESeq2 program was used to identify DEGs in various comparisons, with a filtering criterion of |log2(fold change)| ≥ 0.58 and an FDR < 0.05 [51,52]. GO (Gene Ontology) analysis of the DEGs was performed using the OmicShare biological platform (https://www.omicshare.com/) with a significance level of FDR < 0.05. KEGG (Kyoto Encyclopedia of Genes and Genomes enrichment) analysis was carried out using the OmicShare biological platform with a significance level of <0.05.

### 4.5. qRT-PCR Confirmation of DEGs

RNA samples used for qRT-PCR were the same as those used in RNA-seq analysis. One microgram of total RNA was used for first-strand cDNA synthesis with TransScript^®^ II Multiplex Probe One-Step qRT-PCR SuperMix UDG kit (TransGen Biotech, Beijing, China), following the manufacturer’s instructions. The cDNAs were then used as templates for the qRT-PCR assay. The gene-specific primers used in qRT-PCR are listed in Table S2, and the cotton *GhUBQ7* gene (DQ116441.1) was used as an internal control. The qRT-PCR assays were carried out as mentioned above.

### 4.6. Sugar Content Determination

The leaf samples for sugar content analysis were the same as those used in RNA-seq. Three biological replicates were collected for each time point and treatment. Sugar extraction was carried out according to the reported method [53]. In brief, the freeze-dried materials were crushed using a mixer mill (MM 400, Retsch) with a zirconia bead. In total, 20 mg of powder was extracted with a mixture of methanol/isopropanol/water (3:3:2, *v*/*v*/*v*). The extract was centrifuged at 12,000 rpm at 4 °C for 3 min. Then, 50 μL of the supernatant was evaporated under a nitrogen gas stream and transferred to the lyophilizer for freeze-drying. Then, the dried samples were dissolved in methoxyamine hydrochloride and BSTFA. The dissolved samples were used for GC-MS (Gas Chromatography–Mass Spectrometry, Agilent Technologies, Santa Clara, CA, USA) analysis after dilution to an appropriate concentration.

GC-MS analysis of sugars was performed using an Agilent 8890 gas chromatograph coupled to a 5977B mass spectrometer with a DB-5 MS column (30 m length × 0.25 mm i.d. × 0.25 μm film thickness, J&W Scientific, Agilent Technologies, Santa Clara, CA, USA). Helium was used as a carrier gas at a flow rate of 1 mL/min. Injections were made in the split mode with a split ratio 5:1, and the injection volume was 1 μL. The oven temperature was set at 160 °C (held for 1 min), then raised to 200 °C at 6 °C/min, to 270 °C at 10 °C/min, to 300 °C at 5 °C/min, and to 320 °C at 20 °C/min and held constant for 5.5 min. All samples were analyzed in selective ion monitoring mode. The ion source and transfer line temperatures were 230 °C and 280 °C, respectively.

### 4.7. Data Statistical Analysis

All results shown represent the mean of three independent assays. Statistical analyses were performed using SPSS statistical software version 26.0. A one-way analysis of variance (ANOVA), followed by the T-test with a *p*-value of 0.05, was used to determine significant differences between groups.

## Figures and Tables

**Table 1:** Summary of transcriptome data assembly analysis of X7 and Z2 after infestation by *V. dahliae*.

Sample	Raw Data (Gb)	Clean Data (Gb)	Q20 (%)	Q30 (%)	Proper Map (%)	Unique Map (%)
Z2-48h-M-1	7.37	7.06	98.61	95.39	98.38	94.13
Z2-48h-M-2	7.36	7.07	98.42	94.87	98.26	93.62
Z2-48h-M-3	6.66	6.38	98.36	94.7	98.26	94.15
Z2-48h-T-1	6.59	6.32	98.43	94.92	98.26	92.61
Z2-48h-T-2	7.46	7.4	98.36	94.71	98.44	94.81
Z2-48h-T-3	6.68	6.43	98.47	95.02	98.43	94.34
X7-48h-M-1	6.46	6.19	98.66	95.53	98.48	94.44
X7-48h-M-2	6.44	6.21	98.51	95.11	98.36	94.38
X7-48h-M-3	7.12	6.88	98.47	94.95	98.53	94.51
X7-48h-T-1	7.34	7.03	98.57	95.29	98.53	94.18
X7-48h-T-2	6.62	6.33	98.63	95.48	98.53	94.54
X7-48h-T-3	6.7	6.45	98.47	95.04	98.34	94.24
Z2-72h-M-1	7.11	6.83	98.59	95.35	98.32	94.42
Z2-72h-M-2	7.03	6.77	98.53	95.16	98.33	94.31
Z2-72h-M-3	6.76	6.48	98.56	95.18	98.5	94.48
Z2-72h-T-1	6.52	6.23	98.53	95.2	98.38	94.24
Z2-72h-T-2	7.09	7.02	98.53	95.11	98.47	94.8
Z2-72h-T-3	6.66	6.42	98.58	95.32	98.4	94.3
X7-72h-M-1	7.12	6.79	98.71	95.66	98.43	94.3
X7-72h-M-2	6.48	6.25	98.37	94.78	98.21	94.29
X7-72h-M-3	7.03	6.79	98.3	94.56	98.31	94.36
X7-72h-T-1	7.07	6.8	98.39	94.82	98.32	94.15
X7-72h-T-2	6.25	5.99	98.64	95.51	98.38	94.17
X7-72h-T-3	6.33	6.09	98.41	94.88	98.15	94.04

**Table 2:** HUB genes involved in cotton resistance to *V. dahliae* of each module.

Gene ID	Module Colors	Arabidopsis Homologs	TF Family	Description
GH_A05G0802	pink	AT3G44220	--	NDR1/HIN1-like protein 12
GH_D05G0209	pink	AT1G14920	GRAS	DELLA protein
GH_D09G0664	pink	AT3G06150	--	cytochrome P450 family protein
GH_D12G1284	pink	AT3G61640	--	arabinogalactan protein 20-like
GH_D12G2283	pink	AT5G46910	Jumonji	[histone H3]-trimethyl-L-lysine4 demethylase
GH_A05G3633	light yellow	AT2G29120	--	glutamate receptor, ionotropic, plant
GH_A05G4276	light yellow	AT4G33050	--	IQ domain-containing protein
GH_A09G2426	light yellow	AT3G23000	--	carbon catabolite-derepressing protein kinase
GH_A05G1713	purple	AT1G05170	--	beta-1,3-galactosyltransferase
GH_A12G1647	purple	AT1G12990	--	beta-1,4-mannosyl-glycoprotein beta-1,4-N-acetylglucosaminyltransferase
GH_A13G2562	purple	AT3G53750	--	actin, other eukaryote
GH_D06G0094	purple	AT1G47740	--	deubiquitinase DESI2
GH_D12G1462	purple	AT1G02730	--	1,4-beta-D-xylan synthase
GH_A08G1898	salmon	--	--	--
GH_A10G1896	salmon	AT3G17100	--	transcription factor bHLH147
GH_D05G1945	salmon	AT1G75400	--	RING/U-box superfamily protein
GH_D05G2900	salmon	AT5G04885	--	beta-glucosidase
GH_A01G0658	yellow	--	--	--
GH_A05G2937	yellow	--	--	--
GH_A07G0307	yellow	AT5G58070	--	apolipoprotein D and lipocalin family protein
GH_A10G2412	yellow	AT3G47570	--	LRR receptor-like serine/threonine-protein kinase FLS2
GH_D02G0429	yellow	AT3G22142	--	bifunctional inhibitor/lipid-transfer protein/seed storage 2S albumin superfamily protein
GH_D11G1452	yellow	AT3G24750	--	hypothetical protein
GH_A03G2049	brown	AT1G50010	--	tubulin alpha
GH_A05G2067	brown	AT5G67260	--	cyclin D3, plant
GH_D03G1019	brown	AT1G33265	--	protein FATTY ACID EXPORT 4
GH_D04G0151	brown	AT2G40475	--	hypothetical protein
GH_D07G1220	brown	AT3G11780	--	MD-2-related lipid-recognition protein ROSY1
GH_D11G3723	brown	AT2G36880	--	S-adenosylmethionine synthetase
GH_A02G1796	royal blue	AT2G23150	--	natural resistance-associated macrophage protein 2
GH_A05G1292	royal blue	AT3G16000	--	MAR-binding filament-like protein
GH_A05G2396	tan	AT1G21270	--	LRR receptor-like serine/threonine-protein kinase ERECTA
GH_D01G0514	tan	--	--	--
GH_D01G2158	tan	AT1G21550	--	calcium-binding protein CML
GH_D04G1886	tan	AT1G60890	--	1-phosphatidylinositol-4-phosphate 5-kinase

## Data Availability

The datasets presented in this study can be found in online repositories. The names of the repositories and accession numbers can be found at https://www.ncbi.nlm.nih.gov/geo/ (accessed on 2 October 2024), PRJNA1167873.

## Supplementary Materials

The following supporting information can be downloaded at https://www.mdpi.com/article/10.3390/ijms252413326/s1.
