# Supplementary material for: Transcriptome-Based Gene Modules and Soluble Sugar Content Analyses Reveal the Defense Response of Cotton Leaves to Verticillium dahliae
Source: Int J Mol Sci. 2024 Dec 12;25(24):13326. doi: 10.3390/ijms252413326 (PMC11679845; doi:10.3390/ijms252413326)
Supplement: Supplementary file 1 [file ijms-25-13326-s001.zip › ijms-3317218-supplementary.pdf]

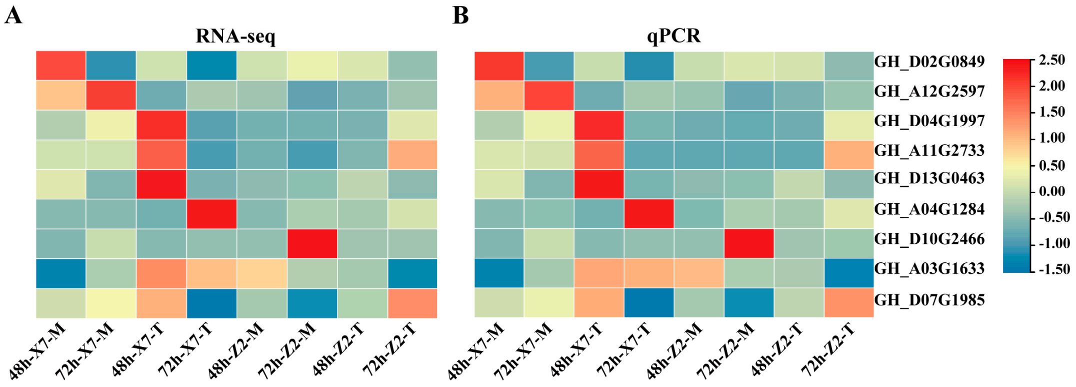

Figure S1. qRT-PCR analysis of RNA-seq results.

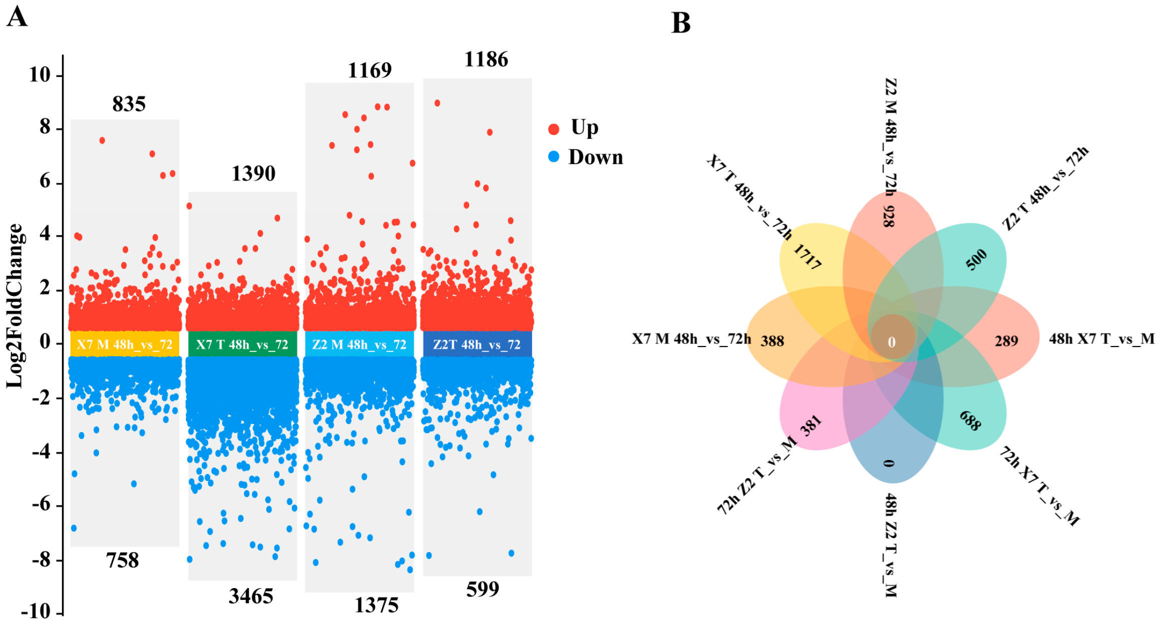

Figure S2. Volcanic plots of DEGs in X7-T vs. Mock and Z2-T vs. Mock comparisons. Red and blue points represent the up-regulated and down-regulated DEGs, respectively.

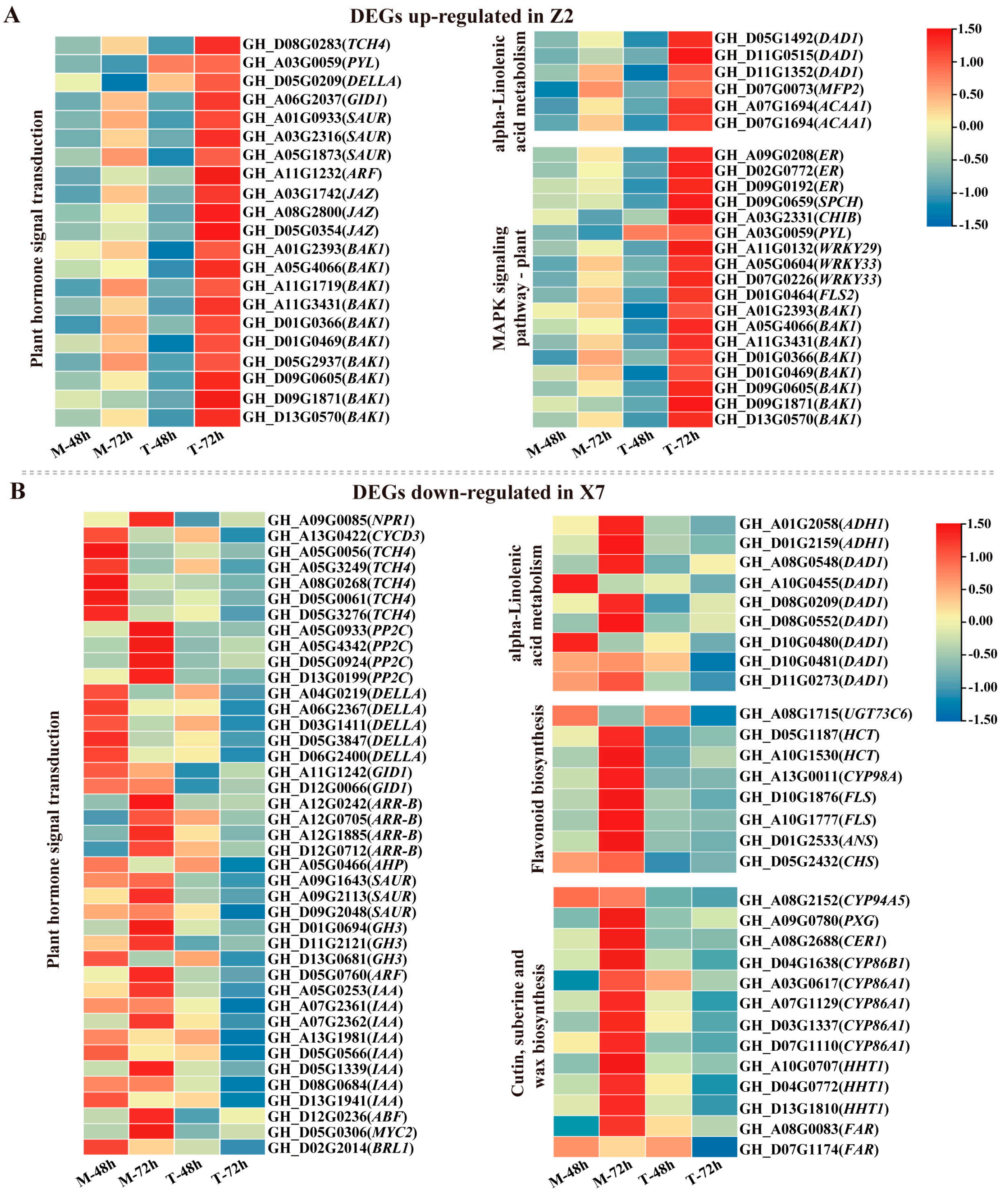

Figure S3: The gene expression heatmap of disease resistance-related pathways in X7 and Z2 after inoculation with *V. dahliae.*

Table S1. DEGs involved in plant-pathogen interactions in modules up-regulated in Z2.

Gene_id	Chr	Start	End	Arabidopsis homologs	Description
GH_A01G0048	A01	306709	353682	AT1G19440	3-ketoacyl-CoA synthase 4
GH_A04G0276	A04	4999800	5002508	AT4G27220	NB-ARC domain-containing disease resistance protein
GH_A05G0604	A05	5525666	5526908	AT1G80840	WRKY DNA-binding protein 40
GH_A05G1725	A05	16293712	16295951	AT1G62300	WRKY family transcription factor
GH_A05G2383	A05	23661883	23662380	AT1G21550	Calcium-binding EF-hand family protein
GH_A05G4014	A05	105129782	105132346	AT3G51550	Malectin/receptor-like protein kinase family protein
GH_A05G4066	A05	106179254	106187337	AT5G60900	receptor-like protein kinase 1
GH_A06G0009	A06	53330	56172	AT1G76040	calcium-dependent protein kinase 29
GH_A07G0368	A07	3783901	3785170	AT4G31800	WRKY DNA-binding protein 18
GH_A07G0986	A07	14281941	14282348	AT2G34930	disease resistance family protein/LRR family protein
GH_A11G0132	A11	1237545	1239221	AT4G23810	WRKY family transcription factor
GH_A11G0476	A11	4181638	4183575	AT2G23770	peptidoglycan-binding LysM domain-containing protein
GH_A11G1719	A11	21396846	21400220	AT1G67000	Protein kinase superfamily protein
GH_A11G3122	A11	111901161	111903635	AT5G60900	receptor-like protein kinase 1
GH_A13G0333	A13	3851955	3852197	AT4G39250	RAD-like 1
GH_D01G0366	D01	3346947	3347921	AT1G70250	receptor serine/threonine kinase
GH_D01G0404	D01	3925431	3929274	AT4G10780	LRR and NB-ARC domains-containing disease resistance protein
GH_D01G0420	D01	4138669	4153640	AT4G26090	NB-ARC domain-containing disease resistance protein
GH_D01G0464	D01	4801874	4806281	AT3G47090	Leucine-rich repeat protein kinase family protein
GH_D01G0469	D01	4927409	4930113	AT5G38260	Protein kinase superfamily protein
GH_D02G2364	D02	68062204	68062938	AT4G39250	RAD-like 1
GH_D03G0092	D03	619401	624176	AT5G66210	calcium-dependent protein kinase 28
GH_D05G1615	D05	13686673	13687260	AT5G47220	ethylene responsive element binding factor 2
GH_D05G2937	D05	29263730	29266334	AT1G66920	Protein kinase superfamily protein

GH_D05G3766	D05	58149542	58150615	AT1G61300	LRR and NB-ARC domains-containing disease resistance protein
GH_D05G4073	D05	514	2258	AT1G53440	Leucine-rich repeat transmembrane protein kinase
GH_D06G1515	D06	46909430	46910041	AT5G47220	ethylene responsive element binding factor 2
GH_D07G0226	D07	2184525	2185513	AT5G26170	WRKY DNA-binding protein 50
GH_D07G2048	D07	49443359	49445590	AT1G62300	WRKY family transcription factor
GH_D07G2134	D07	52168335	52171385	AT1G47890	receptor like protein 7
GH_D09G0605	D09	24731898	24755439	AT5G38260	Protein kinase superfamily protein
GH_D09G1129	D09	37042393	37042947	AT2G14610	pathogenesis-related protein 1
GH_D09G1871	D09	45777762	45787889	AT5G38260	Protein kinase superfamily protein
GH_D09G1934	D09	46508918	46509940	AT4G27950	cytokinin response factor 4
GH_D10G0514	D10	4379089	4390847	AT1G56130	Leucine-rich repeat transmembrane protein kinase
GH_D10G2012	D10	53732362	53733531	AT4G32300	S-domain-2 5
GH_D11G2865	D11	58031716	58035463	AT1G45616	receptor like protein 6
GH_D13G0570	D13	7143294	7145253	AT4G33430	BRI1-associated receptor kinase

Table S2. The primer used in article.

Primer name	Sequences (5'-3')
0463F	AGATGCATCTTCAGGTCCTC
0463R	TGGAGTAATTGATGTAAGGAGA
1524F	ATGCCCTCATCGACATCAATA
1524R	GCTGAACTATTTCCAAAAACATC
1284F	ATGAAGTTCTTCTCTGAATTAGG
1284R	CTCTTTTCCGTAATCTTGCC
1997F	GGGTTAAGAAGACACACATTTA
1997R	TATACGGCGGAGGTTTTGTC
1633F	TGATCATGGAAGGAGATCTAG
1633R	AAAGAAAGAGGTTCCGAAAAC
2597F	CGAAAACCAACAACACCATG
2597R	TTATAATGTCCAGAGCCACCC
0849F	ATGGCTTCTTTTGCTACTTC
0849R	CCATACCAAATGAGATTAACAGA
2466F	AAGACGACTACTGATTTTGATT
2466R	ATGAACAACTTGTTCAATCTCC
1985F	AGCTCAAGGGCAAGCTTTAG
1985R	CCTTGCTTCCTTGACTTGAG
2733F	CGTGAACAACTACTATTTGGTTCC
2733R	AGTGGCACATTGACCACTTG
GhUBQ7F	GAAGGCATTCCACCTGACCAAC

GhUBQ7R	CTTGACCTTCTTCTTCTTGTGCTTG
Ve-ITS1-F	AAAGTTTTAATGGTTCGCTAAGA
ST-VE1-R	CTTGGTCATTTAGAGGAAGTAA
